# The Effect of the Removal of the Area Postrema on Insulin and IGF-1-Induced Cardiovascular and Sympathetic Nervous Responses

**DOI:** 10.1155/EDR.2000.59

**Published:** 2000

**Authors:** Renee Ford, Huiqing Lu, Zhengbo Duanmu, Tadeusz Scislo, Joseph C. Dunbar

**Affiliations:** 1 Department of Physiology Wayne State University School of Medicine 540 E. Canfield Detroit MI 48201-1928 USA

## Abstract

Previous studies have demonstrated that insulin and IGF-1 both increase lumbar sympathetic nerve activity (LSNA) and decrease mean arterial pressure (MAP). We hypothesized that the peripheral responses to insulin and IGF-1 are mediated, at least in part, *via* the central nervous system. In this study we determined the effects of the peripheral administration of both insulin and IGF-1 on cardiovascular dynamics and LSNA following removal of the area postrema (APX), a major site of blood-brain communication. Insulin infusion in normal rats decreased MAP but increased HR and LSNA. When insulin was infused in APX rats it also decreased the MAP but the MAP recovered rapidly and plateaued at a level equivalent to normals after 40 min. Insulin significantly increased the HR and LSNA in the APX rats compared to normals. However, when hypoglycemia was prevented by glucose infusion, the HR and LSNA responses to insulin in the APX rats were similar to normals. IGF-1 also decreased MAP and to a greater extent in the APX rats compared to normals but the increased LSNA in APX rats was equivalent to normals. The APX rats when compared to normals had a greater sensitivity to insulin-induced hypoglycemia while IGF-1 decreased the plasma glucose to a lesser degree in APX rats. We conclude that insulin and IGF-1 entry into the CNS at least *via* the area postrema does not contribute significantly to the hypotensive response and that the greater depressor response to IGF-1 is likely due to enhanced vascular sensitivity in APX rats. The increased HR and LSNA following insulin were likely mediated by an increased reflexive response to hypoglycemia.


*Int. Jnl. Experimental Diab. Res.*, Vol. 1, pp. 59–67
Reprints available directly from the publisher
Photocopying permitted by license only

© 2000 OPA (Overseas Publishers Association) N.V.
Published by license under
the Harwood Academic Publishers imprint,
part of The Gordon and Breach Publishing Group.
Printed in Malaysia.

### The Effect of the Removal of the Area Postrema on Insulin and IGF-1-Induced Cardiovascular and Sympathetic Nervous Responses

RENEE FORD, HUIQING LU, ZHENGBO DUANMU, TADEUSZ SCISLO and JOSEPH C. DUNBAR*

*Department of Physiology, Wayne State University School of Medicine, 540 E. Canfield, Detroit, MI 48201, USA*

*(Received 19 May 1999; Revised 8 September 1999; In final form 28 September 1999)*

**Previous studies have demonstrated that insulin and IGF-1 both increase lumbar sympathetic nerve activity (LSNA) and decrease mean arterial pressure (MAP). We hypothesized that the peripheral responses to insulin and IGF-1 are mediated, at least in part, *via* the central nervous system. In this study we determined the effects of the peripheral administration of both insulin and IGF-1 on cardiovascular dynamics and LSNA following removal of the area postrema (APX), a major site of blood-brain communication. Insulin infusion in normal rats decreased MAP but increased HR and LSNA. When insulin was infused in APX rats it also decreased the MAP but the MAP recovered rapidly and plateaued at a level equivalent to normals after 40 min. Insulin significantly increased the HR and LSNA in the APX rats compared to normals. However, when hypoglycemia was prevented by glucose infusion, the HR and LSNA responses to insulin in the APX rats were similar to normals. IGF-1 also decreased MAP and to a greater extent in the APX rats compared to normals but the increased LSNA in APX rats was equivalent to normals. The APX rats when compared to normals had a greater sensitivity to insulin-induced hypoglycemia while IGF-1 decreased the plasma glucose to a lesser degree in APX rats. We conclude that insulin and IGF-1 entry into the CNS at least *via* the area postrema does not contribute significantly to the hypotensive response and that the greater depressor response to IGF-1 is likely due to enhanced vascular sensitivity in APX rats. The increased HR and LSNA following insulin were likely mediated by an increased reflexive response to hypoglycemia.**

*Keywords:* Area postrema, insulin, IGF-1, sympathetic nervous activity, blood pressure

### INTRODUCTION

Insulin and IGF-1 have homology in its molecular structure and receptor structure as well as metabolic effects. Our laboratory and others have demonstrated that both insulin and IGF-1 act to modulate cardiovascular and sympathetic nervous dynamics where they act to primarily decrease MAP, increase blood flow in selected vascular beds and increase sympathetic nerve activity in rats (Pete *et al.*, 1996; Pete and Dunbar, 1998; Duanmu *et al.*, 1997; Anderson *et al.*, 1991). We have also demonstrated that insulin and IGF-1 injections into the lateral

*Corresponding author. Department of Physiology, Wayne State University School of Medicine, 540 E. Canfield, Detroit, MI 48201-1928, USA. Tel.: (313) 577-1520, Fax: (313) 577-5494, e-mail: jdunbar@med.wayne.edu ventricles can also modulate cardiovascular and sympathetic nervous dynamics (Hu *et al.*, 1996). Based on the above observation we hypothesized that the action of peripheral insulin and IGF-1 on these systems may, in part, be mediated by CNS mechanisms.

The area postrema is a significant site of communication in the blood-brain barrier between the humoral components of the blood and the neural brain tissue (Ferguson and Bains, 1996; Bernstein, 1996). It has been demonstrated that the area postrema is especially significant in mediating responses to nutrients in circulation (Bernstein, 1996; Kenney *et al.*, 1994; Calingasan and Ritter, 1992; Adachi *et al.*, 1991), as well as other factors that mediate autonomic responses (Nakayama *et al.*, 1997; Wade *et al.*, 1996). The area postrema has been demonstrated to be significant in mediating several cardiovascular reflexes such as the response to exercise and baroreflex mechanisms associated with angiotensin II (Bonigut *et al.*, 1997; Cox and Bishop, 1991). Further supporting a role for the area postrema in these processes is the fact that it is juxtaposed and communicates with the nucleus of the solitarius tract (NTS), a major site of cardiovascular regulation (Cai *et al.*, 1996). Additionally, both insulin and IGF have been demonstrated to have abundant receptors at the level of area postrema (Van Houten and Posner, 1981; Nagano *et al.*, 1995). Thus, it is suggested that binding of insulin and IGF-1 to these area postrema receptors mediates regulatory influences on cardiovascular and sympathetic nervous dynamics (Kott *et al.*, 1989).

In previous studies we have proposed that both insulin and IGF-1 levels in circulation alter cardiovascular dynamics by CNS-mediated mechanism (Hu *et al.*, 1996; Wright-Richey *et al.*, 1994; Dunbar *et al.*, 1996). Since the area postrema is one of the significant communicating areas between the brain and periphery, we sought to investigate its role in this process. In this study we evaluated the effects of peripherally administered insulin and IGF-1 on cardiovascular dynamics and sympathetic nerve activity in intact animals compared to animals that had their area postrema removed.

### MATERIALS AND METHODS

Normal male Wistar rats (Harlan Sprague Dawley, Indianapolis, IN) weighing between 285–320 grams were utilized for all studies. Prior to recording studies or following area postrema removal the rats were housed with a 12:12-h light–dark cycle and an ambient temperature of 23°C. The animals were fed laboratory rodent chow and given free access to water. Blood samples were collected and body weights determined. Rats were fasted prior to any experimental recording procedure. Animal studies were conducted in compliance with applicable laws and regulations as well as the principles expressed in the National Institutes of Health, USPHS, Guide for the Care and Use of Laboratory Animals, and the studies were conducted on animals that were lawfully acquired. The Wayne State University Animal Care and Use Committee approved use of animals.

Area postrema removal (APX) was conducted using minor modification of the method of Edwards and Ritter (1981). Briefly, rats were anesthetized with ketamine and xylazine, 80/5 mg/kg (Bayer Corp., Shnunee Mission, KS), placed in a Kopf stereotaxic apparatus, and a dorsal midline incision was made through the skin and epaxial musculature. With the aid of a dissecting microscope, the atlanto-occipital membrane was visualized and punctured, and a portion of it was removed. The area postrema was visualized on the dorsal surface of the medulla at the caudal extent of the fourth ventricle and removed by suction with a 26-gauge needle attached to a vacuum line. With the exception of the vacuum procedure, sham operations were identical to those described for APX rats. The skin was closed and the animals were maintained 3–4 weeks before they were utilized. Following the study, histological sections were prepared to verify the APX.

Measurement of MAP and HR was conducted on animals as described previously following an 18–20 hour fast (Pete and Dunbar, 1998; Duanmu *et al.*, 1997). Briefly, the animals were prepared for cardiovascular recording on a pad heated to maintain the body temperature at 37 ± 1°C. The animals were anesthetized with a mixture of urethane (0.5 g/kg) and $\alpha$-chloralose (80 mg/kg). The trachea was cannulated to minimize respiratory difficulties. Catheters made of PE-50 tubing were filled with heparinized saline (2,000 U/ml) and inserted into the femoral artery to monitor arterial blood pressure *via* a Spectra-Med pressure transducer (Spectra-Med, Oxnard, CA) and into the femoral vein for blood sampling and infusions.

The measurement of sympathetic nerve activity was conducted as previously described (Duanmu *et al.*, 1997) with minor modifications. To measure the sympathetic nerve activity the lumbar sympathetic chain (L3 – L5) was exposed through a midline incision, dissected, cut, and its rostral end placed on stainless steel electrodes for recording of efferent nerve activity. Silicone gel (Wacker Sil-Gel 601A and 601B mixture, Iowa City, IA) was used to embed the nerve bundle and electrodes and allowed to dry and harden for 1 hour. Finally, the abdomen was closed to prevent evaporation. The lumbar sympathetic nerve activity (LSNA) was amplified (5,000 – 10,000 times) and filtered (low at 30 Hz, high at 1,000 Hz) using a Grass RPS 107 amplifier and a Grass IR Z probe (Grass Instruments, Quincy, MA). The amplified and filtered signal was channeled to an oscilloscope HM205. An audio amplifier-loudspeaker (Grass model AM8 audio monitor) was used for auditory monitoring. Whole nerve activity was obtained by rectifying and integrating the action potentials in 1s intervals using a data acquisition system (Dasy Lab Data Acquisition System, Phymed Scientific, Fishers, IN). At the end of each experiment, hexamethonium chloride (20 mg/kg), a ganglionic blocker, was used to determine the relative contribution of pre- and postganglionic fibers to LSNA.

For the experimental recording period normal and APX animals were infused with insulin (5 U; Sigma Corp., St. Louis, MO) or IGF-1 (40 U; Gerelech, Inc., San Francisco, CA) as a bolus and the MAP, HR and LSNA recorded for 1 hour as described above. In some studies to prevent hypoglycemia glucose was infused first as a bolus (45 mg) followed by a constant infusion of 150 mg/kg/hr starting at 15 min before insulin. Blood samples were collected at specified intervals for glucose and/or insulin determination. Glucose determinations were made using a glucose analyzer (YSI; Yellow Spring, OH) and insulin was measured with a double antibody method using a kit (ICN Corp., Costa Mesa, CA).

The data for MAP, HR and LSNA were calculated for individual experiments as percent change from basal (Time 0). The group data were analyzed using ANOVA with repeated measures and/or t-test where appropriate and values expressed as means ± SE.

### RESULTS

The removal of the area postrema in normal rats decreased the body weights initially at two weeks but after 4–5 weeks the body weights and plasma glucose levels were comparable to normals (Tab. I). Also the basal plasma insulin, basal MAP and HR were not different from normals (Tab. I). When we infused insulin as a bolus into normal rats there was a decrease in MAP of approximately 20% after 20 minutes that remained for 60 minutes (Fig. 1A). It can also be observed that bolus insulin infusion into APX rats also resulted in a decrease in MAP that reached a nadir after 20 minutes that was slightly greater than that in normals, but returned to basal levels above normals after 40

TABLE I Plasma glucose and insulin, and basal mean arterial pressure (MAP) and heart rate (HR) in normal rats and in rats 4–5 weeks after removal of the area postrema (APX)

| | Body wt. gm | Glucose mg/dl | Insulin uU/ml | MAP mm Hg | HR beats/min |
|---|---|---|---|---|---|
| Cotrols | 309 ± 12 | 155 ± 78.5 (9) | 36.0 ± 8.8 (9) | 77.0 ± 4 (10) | 365 ± 14 (10) |
| APX | 295 ± 18 | 158 ± 10.1 (9) | 37.0 ± 6.9 (9) | 80.0 ± 6 (10) | 358 ± 13 (10) |

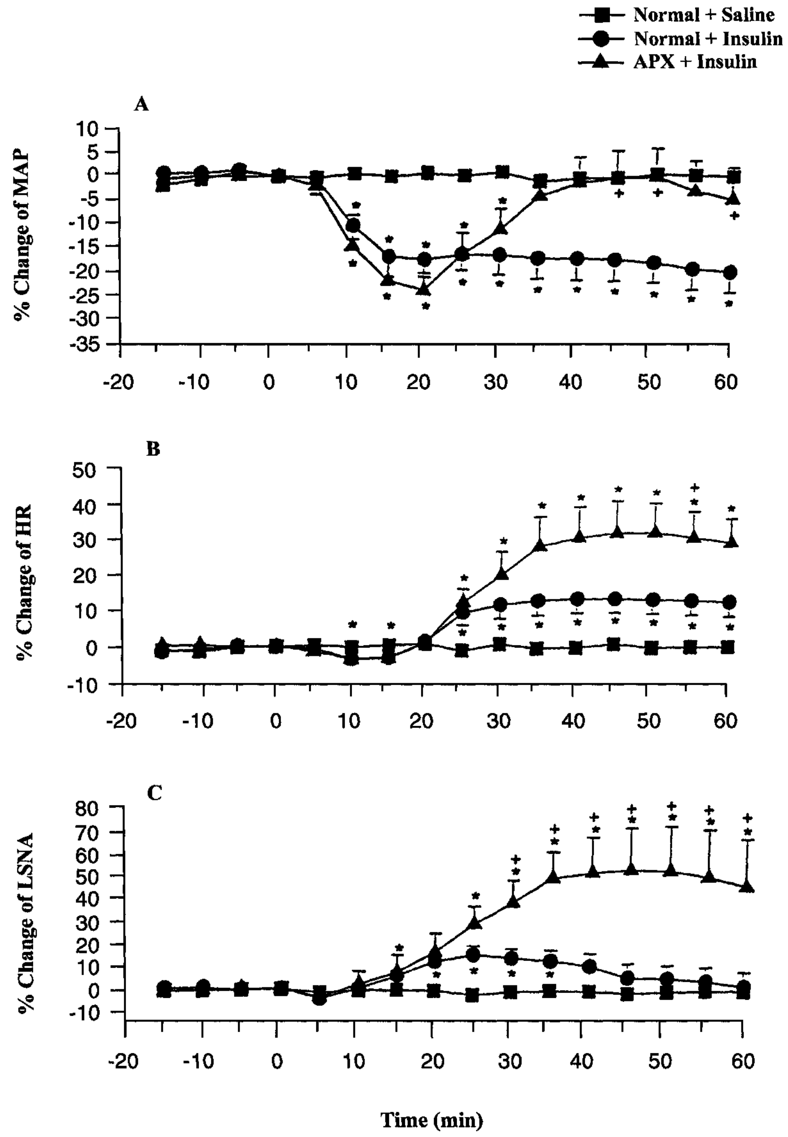

FIGURE 1 The effect of systemic insulin infusion or saline on MAP (Panel A), HR (Panel B) and LSNA (Panel C) in normal and APX rats. Insulin (5 U/animal) significantly decreased the MAP in normal ($P < 0.001$) and APX ($P < 0.001$) rats when compared to saline controls. The HR was significantly increased in normals ($P < 0.001$) and APX ($P < 0.001$) rats. Insulin increased the LSNA in normals ($P < 0.001$) and APX ($P < 0.0001$) rats compared to saline controls. Statistical comparison between groups made by ANOVA with repeated measures. *=significant ($P < 0.05$) differences between normal insulin and APX insulin *versus* normal saline at selected time points. $^{+}$=significant ($P < 0.05$) differences between normal and APX rats at selected time points. $N$=6, 5, 5 for saline controls, normal insulin and APX insulin, respectively.

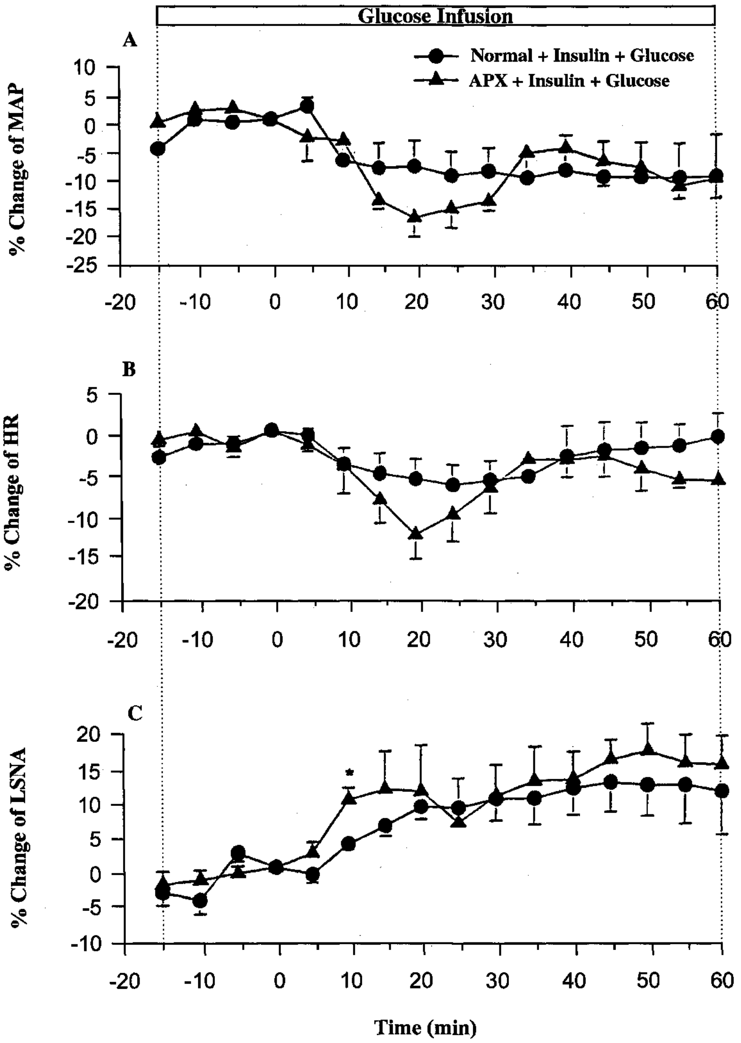

FIGURE 2 The effect of systemic insulin (5 U/animal) infusion as a bolus with previous and subsequent variable glucose infusion (100 mg bolus plus ≅300 mg/hr) to maintain euglycemia on MAP (Panel A), HR (Panel B) and LSNA (Panel C) in normal and APX rats. Insulin significantly decreased the MAP ($P < 0.001$) and HR ($P < 0.001$) and increased the LSNA ($P < 0.001$) in both normal and APX rats. Statistical comparisons between groups made by ANOVA with repeated measures. *=significant ($P < 0.05$) differences between normal and APX rats at selected time points. $N$=5 for both normals and APX animals.

minutes (Fig. 1A). The HR in response to insulin increased approximately 10% in normal animals but the increase in HR was significantly higher (30%) in APX animals when compared to normals (Fig. 1B). The measurement of the lumbar sympathetic nerve activity (LSNA) in these animals demonstrated additionally that bolus insulin infusion increased LSNA approximately 12% in normal animals. This response to insulin was increased to significantly higher levels (approximately 47%) in APX animals (Fig. 1C).

Insulin decreased the blood glucose in APX animals to a greater extent when compared to normals (Tab. II). When these same studies were conducted with glucose infusion to prevent hypoglycemia (plasma glucose maintained above 70 mg/dl), insulin decreased the MAP to a slightly lower level in the APX animals

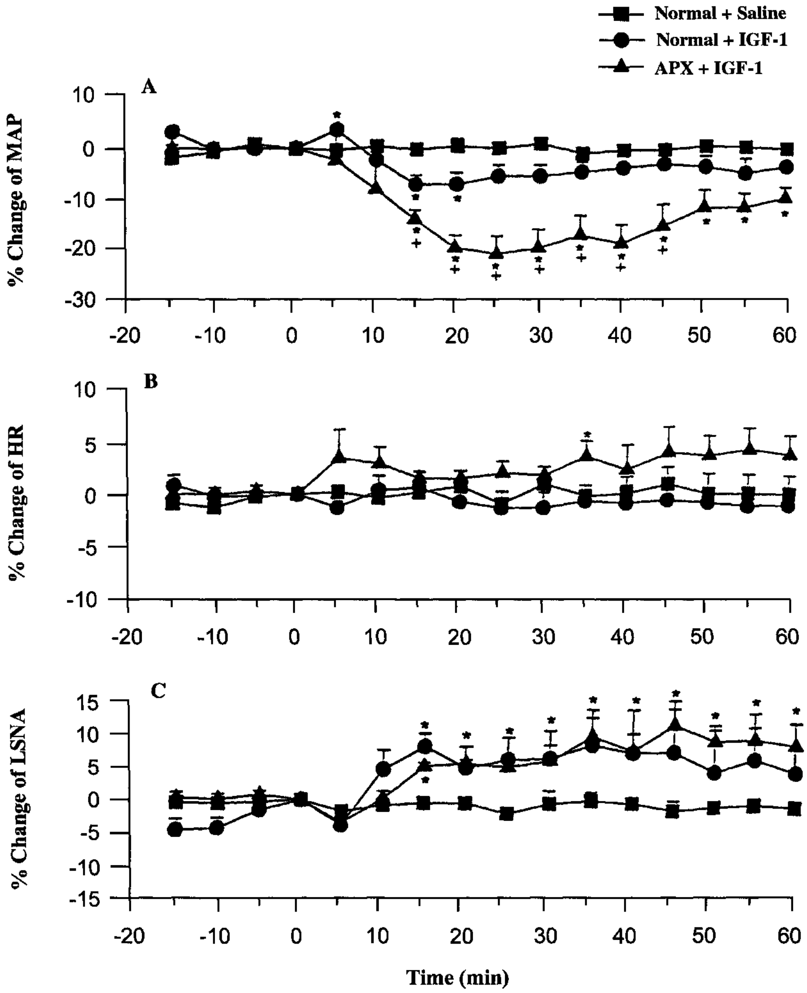

FIGURE 3 The effect of systemic IGF-1 infusion on MAP (Panel A), HR (Panel B) and LSNA (Panel C) in normal and APX rats. IGF-1 (40 ug/animal) significantly decreased the MAP in normals ($P<0.001$) and APX ($P<0.0001$) when compared to saline controls. IGF-1 significantly ($P<0.01$) increased the HR in APX animals compared to controls. IGF-1 significantly increased the LSNA in normal ($P<0.001$) and APX ($P<0.001$) animals. Statistical comparisons between groups made by ANOVA with repeated measures. *=significant ($P<0.05$) differences between normal-IGF-1 and APX-IGF-1 *versus* normal saline at selected time points. $^{+}$=significant ($P<0.05$) differences between normal-IGF-1 and APX-IGF-1 at selected time points. $N$=6, 6, 5 for saline controls, normal IGF-1 and APX IGF-1, respectively.

compared to normals (Fig. 2). When hypoglycemia was prevented the HR was decreased in normal and APX rats and the stimulation of the LSNA was comparable between normal and APX animals (Figs. 2B and C).

When we conducted these same studies using IGF-1, we observed that IGF-1 infusion resulted in an approximately 8% decreased MAP in normal rats and IGF-1 infusion resulted in an even greater decrease (20%) in APX rats (Fig. 3A). IGF-1 did not alter the heart rate in normal but significantly increased it in the APX animals (Fig. 3B). IGF-1 infusion in both normal and APX animals resulted in a significant increase in LSNA activity that was comparable between groups (Fig. 3C).

TABLE II The effect of insulin and IGF-1 infusion on plasma glucose (mg/dl) in normal and APX rats

| | Time (minutes) | | | |
|---|---|---|---|---|
| | 0 | 15 | 30 | 60 |
| Insulin infusion | | | | |
| Normals | 107 ± 4.5<br>(6) | 65 ± 13⁺<br>(6) | 34 ± 16⁺<br>(6) | 33 ± 15⁺<br>(6) |
| APX | 109 ± 10<br>(5) | 46 ± 6.5⁺<br>(5) | 16 ± 2.9*,⁺<br>(5) | 25 ± 3.4⁺<br>(5) |
| Insulin infusion plus glucose | | | | |
| Normals | 130 ± 15 | 90 ± 6⁺ | 98 ± 8⁺ | 100 ± 15 |
| APX | 125 ± 12 | 101 ± 7⁺ | 85 ± 10⁺ | 80 ± 10⁺ |
| IGF-1 infusion | | | | |
| Normals | 114 ± 6<br>(5) | 88 ± 4⁺<br>(5) | 79 ± 5⁺<br>(5) | 68 ± 7⁺<br>(5) |
| APX | 100 ± 5<br>(5) | 97 ± 15<br>(5) | 82 ± 12⁺<br>(5) | 101 ± 14<br>(5) |

* = $P < 0.05$ *vs.* normals.
⁺ = $P < 0.05$ *vs.* values at time 0.

The plasma glucose levels were decreased to a comparable degree in both normal and APX rats in response to IGF-1 infusion (Tab. II).

### DISCUSSION

In this study we have observed that insulin administered peripherally resulted in a sustained decrease in MAP in normals and in animals that had their area postrema removed. However, in APX animals insulin resulted in a slightly greater decrease in MAP but resulted in a more rapid recovery. Our initial hypothesis was that the insulin-induced decrease in MAP was, in part, mediated by insulin entering the CNS *via* area postrema transporters and acting at CNS sites to augment the peripherally mediated decreased MAP. However, the above observations contrastingly support the view that the insulin-mediated cardiovascular responses that decrease MAP are the primary result of direct vascular effects in the periphery (Hausberg *et al.*, 1995; Spraul *et al.*, 1995). In fact, following removal of the area postrema, instead of the anticipated attenuation of the insulin-mediated decrease in MAP, the initial response to insulin was slightly greater, suggesting that the area postrema was important in facilitating not a dilatory but a pressor response. Our observation would be consistent with previous studies that demonstrated that stimulation of the area postrema acted to induce a pressor response (Cai *et al.*, 1996; Hegarty *et al.*, 1995; Suemori *et al.*, 1994). However, this pressor activity was not found in all studies (Gross *et al.*, 1990). In other investigations where the area postrema was removed the animals tended to have lower basal MAP, again suggesting a pressor role of area postrema (Skoog and Mangiapane, 1988). In our study, however, the initial insulin-induced decreased MAP had a more rapid recovery to basal levels in the APX animals. This more rapid recovery was also associated with a greater increase in HR and LSNA. Taken together this suggests that area postrema removal decreased the basal constrictor tone but enhanced the sensitivity to activation of the sympathetic nervous outflow. Our results also suggest that this increased sensitivity to sympathetic activation was likely due to the greater hypoglycemia induced in response to insulin noted in the APX animals. In these animals the blood glucose reached a significantly lower nadir compared to normals that could lead to greater activation of sympathetic counter regulatory outflow (Bonigut *et al.*, 1997; Skoog and Mangiapane, 1988). Although our animals had similar fasting plasma glucose levels, the tendency for lower blood glucose and impaired glucose regulation is consistent with previous observations of decreased caloric regulation (Contreras *et al.*, 1982). Indeed, it has been demonstrated that the counter regulatory responses to changes in plasma nutrients are altered APX animals (Curtis *et al.*, 1996).

Systemic IGF-1 infusion also resulted in a greater decrease in MAP in APX rats when compared to normals. The IGF-1 response supports our previous study which suggests that IGF-1 actions on cardiovascular dynamics are mainly peripheral (Duanmu *et al.*, 1997). The observation that the decreased MAP was greater following IGF-1 with the HR and LSNA responses comparable to normals may be due to the difference in the degree of hypoglycemia that IGF-1 induced. Systemically administered IGF-1 decreased blood glucose to a lesser degree when compared to systemic insulin in both normal and APX animals. Thus, in the absence of the more pronounced hypoglycemia observed in the insulin studies, the enhanced increase in HR and LSNA noted following insulin was not observed.

Nevertheless, this study does not reconcile the observations that insulin and IGF-1 administered into the central nervous system can alter cardiovascular dynamics (Hu *et al.*, 1996; Schultz-Klarr *et al.*, 1994). It may be that the relationship between the insulin and IGF-1 binding sites in the area postrema may result in their translocation to CNS sites at the level of the brainstem, specifically the NTS. These sites may be involved in other physiological regulations such as gastrointestinal function (Carpenter and Briggs, 1986) while the regulation of cardiovascular responses may be mediated at other CNS sites of blood-brain communication such as sites more proximal to the hypothalamus (Ferguson and Bains, 1996).

Thus, even though insulin and IGF-1 bind to receptors in the area postrema and can be demonstrated to translocate to the CNS, we suggest that this process in the area postrema does not appear to be involved in the insulin- or IGF-mediated decreased MAP and increased vascular flow. This study supports a conclusion that insulin and IGF-1 mainly exert their action peripherally to enhance blood flow and decrease MAP.

### Acknowledgement

Supported by NIH GM-08167; NIH MH-47181.

### References

Adachi, A., Kobashi, M., Miyoshi, N. and Tsukamoto, G. (1991) Chemosensitive neurons in the area postrema of the rat and their possible functions, *Brain Res. Bull.*, **26**, 137–140.

Anderson, E. A., Hoffman, R. P., Balon, T. W., Sinkey, C. A. and Mark, A. L. (1991) Hyperinsulinemia produces both sympathetic neural activation and vasodilation in normal humans, *J. Clin. Invest.*, **87**, 2246–2252.

Bernstein, I. L. (1996) Neural mediation of food aversions and anorexia induced by tumor necrosis factors and tumors, *Neurosci. Behav. Rev.*, **20**, 177–181.

Bonigut, S., Bonham, A. C. and Stebbins, C. L. (1997) Area postrema-induced inhibition of the exercise pressor reflexes, *Am. J. Physiol.*, **272**, H1650–H1655.

Cai, Y., Hay, M. and Bishop, V. S. (1996) Synaptic connections and interaction between area postrema and the nucleus tractus solitarius, *Brain Res.*, **724**, 121–124.

Calingasan, N. Y. and Ritter, S. (1992) Hypothalamic paraventricular nucleus lesion do not abolish glucoprivic and lipoprivic feeding, *Brain Res.*, **395**, 25–31.

Carpenter, D. O. and Briggs, D. B. (1986) Insulin excites neurons of the area postrema and causes emesis, *Neurosci. Lett.*, **68**, 85–89.

Contreras, R. J., Fox, E. and Drugovich, M. L. (1982) Area postrema lesions produce feeding deficits in the rat: effects of preoperative dieting and 2-deoxy-D-glucose, *Physiol. Behav.*, **29**, 875–884.

Cox, B. F. and Bishop, V. S. (1991) Neural and humoral mechanisms of angiotensin-dependent hypertension, *Am. J. Physiol.*, **261**, H1284–H1291.

Curtis, K. S., Verbalis, J. S. and Stricker, E. M. (1996) Area postrema lesions in rats appear to disrupt rapid feedback inhibition of fluid intake, *Brain Res.*, **726**, 31–38.

Duanmu, Z., Lapanowski, K. and Dunbar, J. C. (1997) Insulin link growth factor-1 decreases sympathetic nerve activity. The effect is modulated by glycemic status, *Proc. Soc. Exp. Biol. Med.*, **216**, 93–97.

Dunbar, J. C., O'Leary, D. S., Wang, G. and Richey, J. (1996) Mechanisms mediating the insulin-induced hypotension in rats: A role for nitric oxide and autonomic mediators, *Acta Diabetol.*, **33**, 263–268.

Edwards, G. L. and Ritter, R. C. (1981) Ablation of the area postrema causes exaggerated consumption of preferred food in the rat, *Brain Res.*, **216**, 265–276.

Ferguson, A. V. and Bains, J. S. (1996) Electrophysiology of the circumventricular organs, *Front. Neuroendocrinol.*, **17**, 440–475.

Gross, P. M., Wainman, D. S., Shover, S. W., Wall, K. M. and Ferguson, A. V. (1990) Metabolic activation of efferent pathways from the rat area postrema, *Am. J. Physiol.*, **258**, R788–R797.

Hausberg, M., Mark, A. L., Hoffman, R. P., Sinkey, C. A. and Anderson, E. A. (1995) Dissociation of sympathoexcitatory and vasodilatory actions of modesty elevated plasma insulin levels, *J. Hypertens.*, **13**, 1015–1021.

Hegarty, A. A., Hayward, L. F. and Felder, R. B. (1995) Sympathetic response to stimulation of area postrema in decerebrate and anesthetized rats, *Am. J. Physiol.*, **268**, H1086–H1095.

Hu, Y., Pete, G., Walsh, M. F. and Dunbar, J. C. (1996) Central IGF-1 decreases systemic blood pressure and increases blood flow in selective vascular beds, *Horm. Metab. Res.*, **28**, 211–214.

Kenney, N. J., Tomoyasu, N. and Burkhart, M. K. (1994) Food aversion induced by area postrema ablation, *Appetite*, **22**, 205–220.

Kott, J. N., Kenney, N. J., Bhatia, A. J. and Bhatia, A. M. (1989) Response to chronic insulin administration: effect of area postrema ablation, *Physiol. Behav.*, **46**, 971–976.

Nagano, T., Sato, M., Mori, Y., Du, Y., Takagi, H. and Tohyama, M. (1995) Regional distribution of messenger RNA encoding the insulin-like growth factor Type 2 receptor in the rat lower brain stem, *Brain Res. Mol. Brain Res.*, **32**, 14–24.

Nakayama, Y., Takano, Y., Eguchi, K., Migita, K., Saito, R., Tsujimoto, G. and Kamiya, H. (1997) Modulation of the arterial baroreceptor reflex by the vasopressin receptor in the area postrema of the hypertensive rats, *Neurosci. Lett.*, **226**, 179–182.

Pete, G. and Dunbar, J. C. (1998) Regional blood flow dynamics in response to insulin and IGF-1 in diabetic animals, *Clin. Exp. Hypertens.*, **20**, 67–83.

Pete, G., Hu, Y., Walsh, M. F., Sowers, J. and Dunbar, J. C. (1996) IGF-1 decreases mean blood pressure and selectively increases regional blood flow in normal rats, *Proc. Soc. Biol. Med.*, **213**, 187–192.

Schultz-Klarr, S., Wright-Richey, J. and Dunbar, J. C. (1994) The effect of systemic *versus* intracerebroventricular administration of insulin, 2-deoxyglucose and glucose on metabolic and cardiovascular responses, *JAAMP*, **5**, 152–158.

Skoog, K. M. and Mangiapane, M. L. (1988) Area postrema and cardiovascular regulation in rats, *Am. J. Physiol.*, **254**, H963–H969.

Spraul, M., Rauussin, E. and Baron, A. D. (1995) Lack of relationship between muscle sympathetic nerve activity and skeletal muscle vasodilation in response to insulin infusion, *Diabetologia*, **39**, 91–96.

Suemori, K., Kobashi, M. and Adachi, A. (1994) Effect of gastric distension and electrical stimulation of dorsal medial medulla on neurons in parabrachial nucleus of rats, *J. Auton. Nerv. Sys.*, **48**, 221–229.

Van Houten, M. and Posner, B. I. (1981) Specific binding and internalization of blood-borne [125 I]-iodoinsulin by neurons of the rat area postrema, *Endocrinology*, **109**, 853–859.

Wade, G. N., Schneider, J. E. and Li, H. Y. (1996) Control of fertility by metabolic cues, *Am. J. Physiol.*, **270**, E1–E19.

Wright-Richey, J., Schultz-Klarr, S. and Dunbar, J. C. (1994) The effect of ventral medial hypothalamic lesion on the insulin-induced hypotensive response in normal rats, *Acta Diabetol.*, **31**, 91–97.

